# Detecting delirium: a systematic review of ultrabrief identification instruments for hospital patients

**DOI:** 10.3389/fpsyg.2023.1166392

**Published:** 2023-05-12

**Authors:** Yadong Liu, Zhenzhen Li, Ying Li, Ning Ge, Jirong Yue

**Affiliations:** ^1^Department of Geriatrics and National Clinical Research Center for Geriatrics, West China Hospital/West China School of Medicine, Sichuan University, Sichuan, China; ^2^Health Management Center, General Practice Center, National Clinical Research Center for Geriatrics, West China Hospital, Sichuan University, Sichuan, China

**Keywords:** delirium, measurement, systematic review, psychometrics, older patients

## Abstract

**Objective:**

Early identification of delirium, which often occurs in older patients, can effectively reduce adverse prognoses. One way to increase the detection rate of delirium is to use an effective ultrabrief instrument for higher-frequency screening. The purpose of this review is to evaluate the diagnostic accuracy of ultrabrief screening tools for delirium.

**Methods:**

The Cochrane Library, PubMed and EMBASE were searched from January 1, 1974, to November 31, 2022. We assessed the measurement properties of screening instruments using the consensus-based standards for selecting health measurement instruments (COSMIN) checklist and evaluated the risk bias of the included studies using the Quality Assessment of Diagnostic Accuracy Studies-2 (QUADAS-2) tool. The diagnostic test accuracy of instruments for delirium was reported using sensitivity, specificity, positive likelihood ratio (PLR) and negative likelihood ratio (NLR).

**Result:**

Of the 4,914 items identified, 26 met the eligibility criteria, resulting in 5 different delirium identification tools. The overall study quality assessed by the QUADAS-2 tool was moderate to good. Of the five screening tools, two instruments had sensitivity ≥80% and specificities ≥80%: 4AT and UB-2. The most comprehensive is the 4AT scale, which has a sensitivity of 0.80 [95% confidence interval (CI):0.68, 0.88] and a specificity of 0.89 (95%CI: 0.83, 0.93) and contains 4 items. UB-2 has a sensitivity of 0.88 (95%CI: 0.72, 0.96) and a specificity of 0.64 (95%CI: 0.56, 0.70).

**Conclusion:**

UB-2 and MOTYB had excellent sensitivity for delirium screening at an early stage. In terms of sensitivity and intentionality, the 4AT is the best recommended scale.

## Introduction

Delirium is the clinical manifestation of acute encephalopathy, which is characterized by acute disorders of consciousness, attention, and cognition that fluctuate over time and are fundamental criteria in delirium diagnosis (Oh et al., 2017). It is a common disease that affects many hospitalized patients, especially those aged 65 and over. Prolonged hospitalization and decreased cognitive ability are considered risk factors for delirium, while delirium itself is a known complication of dementia and is associated with an increased risk of death (Breitbart et al., 2002). Many cases of delirium are not recognized, which means that the opportunity for prevention has been lost (MacLullich and Hall, 2011). Early detection is helpful for treatment and could reduce the duration and adverse effects of delirium. Although delirium screening is the standard procedure in many hospitals, up to 72% of delirium events have not been found or misdiagnosed (de la Cruz et al., 2015). The failure may be due to the fluctuation of delirium symptoms. The patient may not have developed delirium at routine screening. Therefore, it is particularly important to screen for delirium multiple times per day or every day, as well as obtain collateral history from a reliable caregiver, to detect its fluctuating nature.

At present, there are more than 40 delirium instruments for different purposes (e.g., screening, diagnosis and severity), for different clinical environments (e.g., intensive care units, emergency departments and medical wards), and for different users (e.g., psychiatrists, geriatricians, nurses, and caregivers; Helfand et al., 2021). Such a large number of instruments not only makes the direct comparison of evaluation results challenging but also increases the difficulty of selecting instruments for clinical staff. To detect delirium more efficiently, it is best to use a simple and rapid instrument to screen delirium. We named this rapid delirium screening instrument with an evaluation time ≤2 min and a number of items ≤4 the ultrabrief delirium screening instrument. This means that they can be routinely used 2–3 times a day in clinical situations. Thus, the recognition of delirium by clinical staff can be improved.

At present, many delirium screening scales are committed to simplifying and improving delirium detection. The MOTYB (the months of the year backwards test) is a commonly used attention test (Ryan et al., 2018). The 4 ‘A’s test or 4AT is a short delirium assessment tool intended for clinical use in general settings when delirium is suspected and was initially published on a dedicated website in 2011 (Bellelli et al., 2014). UB-2 (ultrabrief screen), consisting of the two most sensitive items in the 3 min diagnostic CAM (3D-CAM) (Fick et al., 2015), was used recently and shown to be useful in delirium screening. While many systematic reviews of delirium instruments exist, they all focus on a certain instrument or comprehensive evaluation (Wong et al., 2010; Morandi et al., 2012; LaMantia et al., 2014; De and Wand, 2015; Jeong et al., 2020; Helfand et al., 2021). However, to the best of our knowledge, no systematic reviews have comprehensively compared the diagnostic accuracy between those different ultrabrief delirium screening instruments.

The objective of this review is threefold. First, we assessed the measurement properties of screening instruments using the consensus-based standards for selecting consensus-based standards for the selection of health status measurement instruments (COSMIN) checklist (Mokkink et al., 2009). Second, we evaluated the risk bias of study quality using the Quality Assessment of Diagnostic Accuracy Studies-2 (QUADAS-2) tool. Third, we examined the diagnostic accuracy of ultrabrief delirium screening instruments in various care settings. The findings of this investigation provide recommendations for the choice of ultrabrief screening tools for delirium.

## Materials and methods

### Literature search strategy

Two authors conducted independent literature searches. The Cochrane Library, PubMed and EMBASE were searched from January 1, 1974, to November 31, 2022. Studies were included when they met the following criteria: (1) reported at least one delirium screening instrument; (2) examination of diagnostic accuracy against a widely accepted diagnostic criterion of delirium, such as the Diagnostic and Statistical Manual of Mental Disorders (DSM, Version III, IV or V), the International Classification of Diseases (ICD), or recognized instruments for delirium assessment, such as the confusion assessment method (CAM) and delirium rating scale (DRS). Exclusion criteria were: (1) case series, comments, letters, protocol, meeting reports; (2) non-English-language publications; (3) studies on delirium in children; (4) the scales involved in the study do not meet the requirements that the average use time is ≤2 min and the number of items is ≤4. The search terms included the keywords “delirium” and “instrument,” as well as their known synonyms. The detailed search strategy is shown in the Supplementary material (supplement 1).

### Study selection and data extraction

Two independent authors (YaL and ZL) screened the relevant literature by title and abstract and then read the full text to select eligible articles. Any disagreement was resolved by consulting a third author (JY). We collected the following information: sample size, language, study design, study sites, country, application of reference standard and examiner specialty. We also calculated/extracted the sensitivity, specificity, area under the ROC curve (AUC), and other diagnostic accuracy indices of each study.

### Risk of bias assessment

Two independent review authors (YaL and ZL) assessed the methodological quality of the studies using the Diagnostic Accuracy Study Quality Assessment (QUADAS-2) tool. This tool is available at https://www.bris.ac.uk/quadas. The QUADAS-2 tool assessed the study quality from four aspects: participant selection, index test, reference standards, flow and timing. Differences were resolved by a third author (JY).

### Measurement property assessment

We used the COSMIN guidelines to rate the measurement properties for each delirium screening instrument. The COSMIN checklist is a tool for assessing the reliability and validity of the screening instrument, which is available at https://www.cosmin.nl. We evaluated the screening instrument from six aspects: (1) content validity; (2) structural validity; (3) reliability; (4) internal consistency; (5) cross-cultural validity; and (6) criterion validity. We reviewed all relevant articles about each instrument to make an accurate decision. The ratings on each of the COSMIN criteria were summed and reported as a 0 to 6 score (Appendix 2) using an adaptation of the COSMIN scoring procedure published previously (Helfand et al., 2021). For reporting on each of these categories, the instruments were given one point; failure to report on these categories resulted in no points. Two authors carefully extracted information from each article according to the COSMIN framework.

### Statistical analysis

Meta-analyses were performed using the Stata (version 16.0, StataCorp, TX, United States) MIDAS module. Sensitivity, specificity, positive likelihood ratio (PLR), negative likelihood ratio (NLR), and area under the curve (AUC) were used to report diagnostic test accuracy for delirium instruments. Sensitivity, specificity, and likelihood ratios were calculated from the raw data and then rounded for display in the data tables. In general, larger PLRs and smaller NLRs indicate better diagnostic performance. AUC ≥0.9 indicates high diagnostic accuracy, 0.7–0.9 indicates moderate diagnostic capability, and 0.5–0.7 indicates low accuracy.

Heterogeneity was divided into low, moderate, and high with *I*^2^ values of 25%, 50%, and 75%, respectively. To explore the sources of heterogeneity, we performed a subgroup analysis for different sites (ICU or non-ICU). To investigate the robustness we found, we performed sensitivity analyses. We analysed only DSM standard studies. We evaluated the publication bias of all eligible studies using Deek’s funnel plot.

## Results

### Selection process

**Figure 1:**
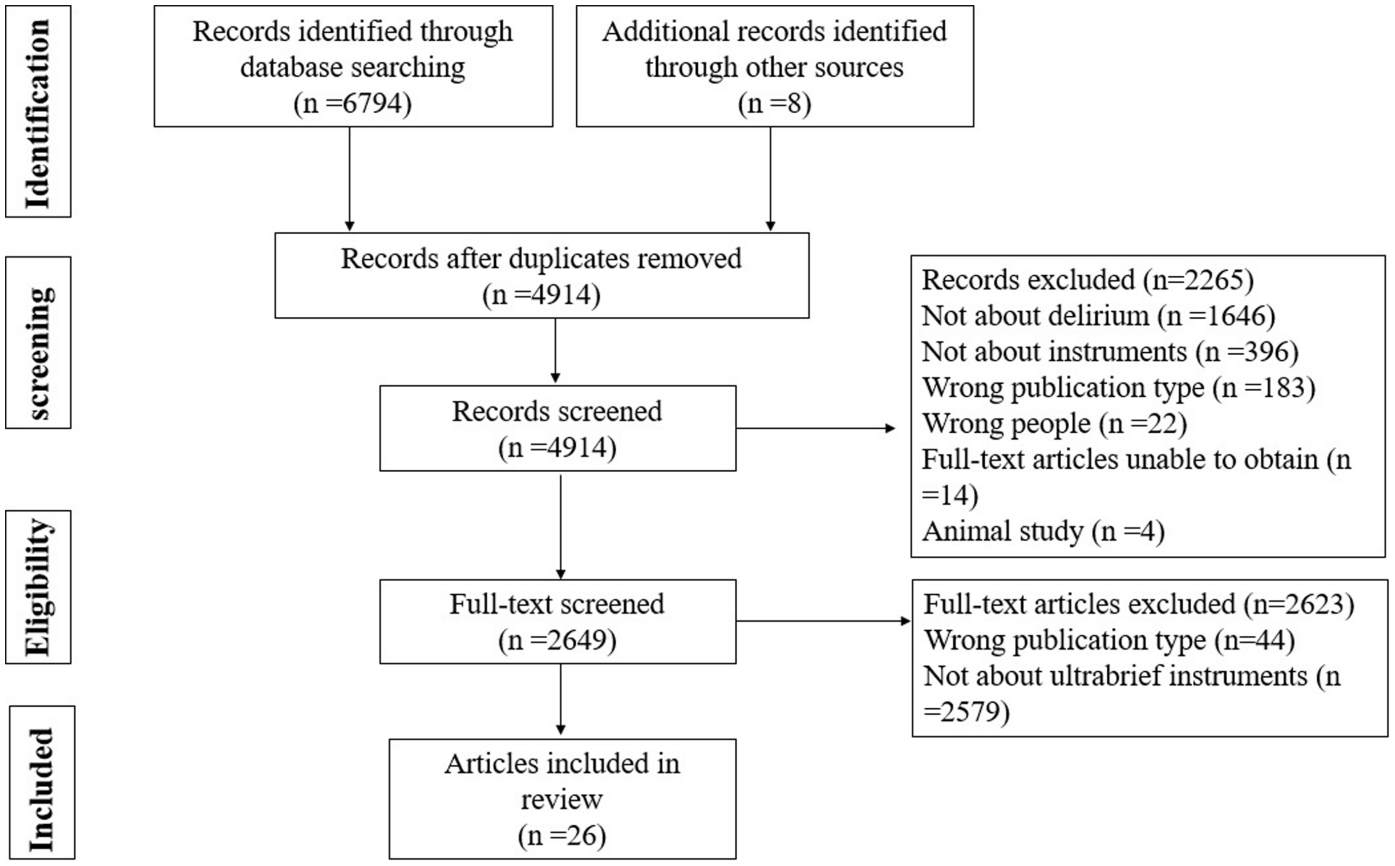
PRISMA flow chart diagram of the study selection process.

Figure 1 displays the PRISMA flowchart of the literature search and selection. We retrieved 4,914 potentially relevant records. A total of 2,265 records were excluded after title and abstract screening. Finally, 2,649 full texts were screened, of which 26 articles reporting five delirium screening instruments met the eligibility criteria and were included in this review. Five screening tools are 4AT (Robson et al., 2017), MOTYB (Marra et al., 2018), O3DY (Bédard et al., 2019), AMT-4 (Swain and Nightingale, 1997) and UB-2 (Fick et al., 2015).

### Study characteristics

Table 1 shows the characteristics of all 26 included studies. A total of 7,262 participants were included. Eight studies (30.8%) were developed in ICUs, 3 studies (11.5%) were developed in stroke units, and 15 studies (57.7%) were conducted in non-ICUs. The gold standards used in each of the 26 articles include DSM (46.2%), CAM (50%), and DRS (3.8%).

**Table 1:** Characteristics of the included studies and main findings.

Source	Study design	Study site	Country/Language	Sample size	Examiner specialty	Delirium prevalence, %	Reference standard	Sensitivity	Specificity	PLR	NLR
*4AT*											
Asadollahi (2016)	Cross-sectional study	Nursing homes and daily care centers	Iran/Persian	293	Nurse	56	DSM	0.35 (0.28–0.43)	0.97 (0.92–0.99)	11.21 (4.18–30.08)	0.67 (0.60–0.76)
Bellelli et al. (2014)	Prospective consecutive patient study	Acute geriatric and rehabilitation wards	Italy/Italian	234	Expert assessors	12.4	DSM	0.90 (0.73–0.98)	0.84 (0.78–0.89)	5.62 (4.02–7.87)	0.12 (0.04–0.36)
Myrstad et al. (2019)	Retrospective, quality improvement study	Acute geriatric wards	Norway/Norwegian	49	Nurse	42.8	DSM	0.50 (0.27–0.73)	0.86 (0.68–0.96)	3.63 (1.32–9.95)	0.58 (0.37–0.92)
Casey et al. (2019)	Prospective study	Multi-site health service	Australia/English	559	Nurse	16.3	CAM	0.65 (0.54–0.75)	0.90 (0.87–0.92)	6.32 (4.65–8.60)	0.39 (0.30–0.92)
MacLullich et al. (2019)	Prospective, double-blind diagnostic test accuracy study	Emergency departments or in acute general medical wards	UK/English	392	Expert assessors	–	CAM	0.76 (0.61–0.87)	0.94 (0.91–0.97)	13.63 (8.56–21.71)	0.26 (0.16–0.42)
Kuladee and Prachason (2016)	Cross-sectional study	General medical wards	Thailand/Thai	97	Psychiatrist	24.7	CAM	0.83 (0.63–0.95)	0.86 (0.76–0.93)	6.08 (3.33–11.12)	0.19 (0.08–0.47)
Hendry et al. (2016)	Prospective consecutive patient study	Geriatric hospital wards	UK/English	434	Clinician	18.6	CAM	0.87 (0.78–0.93)	0.70 (0.64–0.74)	2.85 (2.38–3.40)	0.19 (0.11–0.33)
De et al. (2017)	Prospective study	Geriatric and orthogeriatric hospital wards	Australia/English	257	Expert assessors	61.9	DSM	0.87 (0.81–0.92)	0.80 (0.70–0.87)	4.25 (2.86–6.32)	0.17 (0.11–0.25)
Gagné et al. (2018)	Prospective study	Emergency departments	Canada/French	319	Expert assessors	15.4	CAM	0.90 (0.78–0.97)	0.60 (0.54–0.66)	2.24 (1.89–2.67)	0.17 (0.07–0.39)
O’Sullivan et al. (2018)	Prospective nonconsecutive study	Emergency departments	Ireland/English	350	Clinician	11	DSM	0.93 (0.83–0.98)	0.91 (0.88–0.94)	10.87 (7.43–15.92)	0.08 (0.03–0.19)
Saller et al. (2019)	Prospective consecutive study	Recovery room	Germany/German	543	Expert assessors	10.5	CAM	0.95 (0.77–1.00)	0.99 (0.98–1.00)	124.33 (46.64–331.42)	0.05 (0.01–0.31)
Infante et al. (2017)	Prospective and Cross-sectional study	Stroke units	Italy/Italian	100	Neurologist	52	DSM	0.96 (0.96–1.00)	0.76 (0.62–0.87)	4.00 (2.43–6.57)	0.05 (0.01–0.21)
Lees et al. (2013)	Prospective consecutive study	Stroke units	UK/English	100	Nurse	11	DSM	1.00 (0.74–1.00)	0.82 (0.72–0.87)	5.19 (3.31–8.12)	0.05 (0.00–0.72)
Shenkin et al. (2019)	Prospective study	Emergency room and acute geriatric wards	UK/English	395	Nurses or trained associates	12.4	CAM	0.45 (0.35–0.56)	0.96 (0.93–0.98)	10.45 (5.87–18.58)	0.57 (0.48–0.69)
Koca et al. (2022)	Cross-sectional study	Hospital	Turkey/Turkish	123	Nurse	13.8	DSM	0.67 (0.41–0.87)	0.94 (0.88–0.98)	11.67 (5.02–27.10)	0.35 (0.18–0.68)
Johansson et al. (2021)	Cross-sectional study	Hospital	Swedish/Swiss	159	Expert assessors	19	DSM	0.43 (0.23–0.66)	0.81 (0.73–0.87)	2.27 (1.27–4.06)	0.70 (0.48–1.01)
*AMT-4*											
Hendry et al. (2016)	Prospective consecutive patient study	Geriatric hospital wards	UK/English	408	Clinician	18.6	CAM	0.93 (0.85–0.97)	0.54 (0.48–0.59)	2.00 (1.75–2.28)	0.14 (0.06–0.30)
Lees et al. (2013)	Prospective consecutive study	Stroke Units	UK/English	111	Nurse	11	DSM	0.83 (0.52–0.98)	0.55 (0.44–0.65)	1.83 (1.31–2.56)	0.31 (0.09–1.10)
Dyer et al. (2017)	Cross-sectional study	Emergency departments	Germany/German	196	Research assistants	26	CAM	0.92 (0.75–0.99)	0.82 (0.75–0.87)	5.06 (3.61–7.09)	0.09 (0.02–0.36)
*MOTYB*											
Hendry et al. (2016)	Prospective consecutive patient study	Geriatric hospital wards	UK/English	406	Clinician	18.6	CAM	0.91 (0.83–0.96)	0.50 (0.44–0.55)	1.81 (1.60–2.06)	0.18 (0.09–0.36)
Marra et al. (2018)	Prospective observational study	Emergency departments	US/English	235	Clinician	10.6	DSM	0.84 (0.64–0.95)	0.52 (0.45–0.59)	1.75 (1.40–2.18)	0.31 (0.12–0.76)
O’Regan et al. (2017)	Cross-sectional study	Hospital	UK/English	440	Expert assessors	39	DRS	0.85 (0.78–0.90)	0.58 (0.52–0.64)	2.04 (1.75–2.37)	0.26 (0.18–0.39)
Voyer et al. (2016)	Cross-sectional study	Acute care hospital and LTC facility	Canada/English	191	Expert assessors	12	CAM	0.83 (0.61–0.95)	0.38 (0.30–0.45)	1.32 (1.06–1.65)	0.46 (0.19–1.15)
O’Regan et al. (2014)	Cross-sectional study	Hospital	UK/English	265	Expert assessors	19.6	DSM	0.83 (0.70–0.93)	0.91 (0.86–0.94)	9.04 (5.84–13.99)	0.27 (0.17–0.44)
*O3DY*											
Bédard et al. (2019)	Cross-sectional study	Emergency departments	French/French	313	Expert assessors	6	CAM	0.84 (0.75–0.91)	0.58 (0.52–0.64)	2.01 (1.71–2.37)	0.27 (0.17–0.44)
*UB-2*											
Marcantonio et al. (2022)	Prospective cohort study	Hospital	US/English	293	Certified nursing assistants	22	CAM	0.35 (0.28–0.43)	0.97 (0.92–0.99)	11.21 (4.18–30.08)	0.67 (0.60–0.76)

4AT, 4 attention test; AMT-4, 4-point abbreviated mental test; MOTYB, months of the year recited backwards; O3DY, Ottowa day, date, WORLD BW and year; UB-2, ultra-brief 2-item screen; DSM, diagnostic and statistical manual of mental disorders; CAM, confusion assessment method; DRS, delirium rating scale. PLR, positive likelihood ratio; NLR, negative likelihood ratio.

### Study quality assessed By The QUADAS-2 tool

Table 2 summarizes the study quality risk biases assessed by the QUADAS-2 tool. The overall risk of bias was rated as low to moderate. Eight studies were considered to have low-risk bias. Fourteen studies were rated as having a high risk. Potential biases for our systematic review were listed as follows: (1) participant selection (e.g., ICU or non-ICU patients); (2) secondary analysis of retrospective studies was also considered high risk. The retrospective design may have introduced selection bias.

**Table 2:** Risk bias of included studies by the QUADAS-2 tool.

	Risk of bias				Applicability concerns		
	Patient selection	Index test	Reference standard	Flow and timing	Patient selection	Index test	Reference standard
Asadollahi (2016)	?	?	?	h	?	?	?
Bellelli et al. (2014)	l	?	l	l	l	?	l
Myrstad et al. (2019)	h	?	?	h	h	?	?
Casey et al. (2019)	l	l	l	l	l	l	l
MacLullich et al. (2019)	?	l	l	?	?	l	l
Kuladee and Prachason (2016)	?	l	l	l	?	l	l
Hendry et al. (2016)	l	?	l	l	l	?	l
De et al. (2017)	l	l	l	l	l	l	l
Gagné et al. (2018)	h	?	?	?	h	?	?
O’Sullivan et al. (2018)	l	?	l	l	l	?	l
Saller et al. (2019)	l	l	l	l	l	l	l
Infante et al. (2017)	h	?	?	?	h	?	?
Lees et al. (2013)	l	l	l	?	l	l	l
Shenkin et al. (2019)	l	l	l	l	l	l	l
Koca et al. (2022)	l	l	l	l	l	l	l
Johansson et al. (2021)	l	l	l	l	l	l	l
Hendry et al. (2016)	l	?	l	l	l	?	l
Lees et al. (2013)	l	l	l	?	l	l	l
Dyer et al. (2017)	l	l	?	?	l	l	?
Hendry et al. (2016)	l	?	l	l	l	?	l
Marra et al. (2018)	l	l	l	l	l	l	l
O’Regan et al. (2017)	l	l	l	l	l	l	l
Voyer et al. (2016)	l	l	l	h	l	l	l
O’Regan et al. (2014)	l	l	l	l	l	l	l
Bédard et al. (2019)	l	l	l	l	l	l	l
Marcantonio et al. (2022)	l	?	?	l	l	?	?

h, high risk; l, low risk; ?, uncertain.

### COSMIN assessment of screening instruments

We used the COSMIN standards to assess the psychometric properties (reliability and validity) of five screening tools. We chose the single earliest publication for each instrument. The summarized COSMIN assessment results are shown in Table 3. None of the included studies reported internal reliability. All five instruments have internal consistency and effect indicators. The 4AT and MOTYB have good content validity. The AMT-4 and UB-2 have adequate construct validity. For external validity, the MOTYB is the only one that lacks it.

**Table 3:** COSMIN checklist of screening instruments.

Scale	Effect indicators	Content validity	Internal consistency	Interrater reliability	Construct validity	External validity*
4AT	+	+	+	−	−	+
MOTYB	+	+	+	−	−	−
O3DY	+	+	−	−	−	+
AMT-4	+	+	−	−	+	+
UB-2	+	+	−	−	+	+

+, have this item; −, does not have this item.

### Diagnostic accuracy of screening tools

Studies have reported data on the diagnostic accuracy of all five screening tools for delirium: the 4AT, the MOTYB, the AMT-4, the O3DY, and the UB-2 (Table 3).

The 4AT (*n* = 16 studies) had a pooled sensitivity of 80% [95% confidence interval (CI): 68%–88%] and a pooled specificity of 89% (95% CI: 83%–93%); the pooled PLR and NLR were 7.3 (95% CI: 4.7–11.4) and 0.23 (95% CI: 0.14–0.37), respectively. The pooled estimates of sensitivity and specificity for the MOTYB (*n* = 5 studies) were 87% (95% CI: 83%–90%) and 61% (95% CI: 44%–76%), respectively; the pooled PLR and NLR were 2.2 (95% CI: 1.5–3.4) and 0.22 (95% CI: 0.15–0.30), respectively. The AMT-4 had a sensitivity of 93% [95% CI: 85%–97%] and a specificity of 54% (95% CI: 48%–59%); the O3DY had a sensitivity of 84% [95% CI: 75%–91%] and a specificity of 58% (95% CI: 52%–64%); and the UB-2 had a sensitivity of 88% [95% CI: 72%–96%] and a specificity of 61% (95% CI: 44%–76%). More details, such as the pooled PLR and NLR, are shown in Table 4.

**Table 4:** Summary estimates of pooled diagnostic accuracy.

Instrument	Study (sample)	Pooled sensitivity (95% CI)	Pooled specificity (95% CI)	Pooled PLR (95% CI)	Pooled NLR (95% CI)
4AT	16 (4404)	0.80 (0.68, 0.88)	0.89 (0.83, 0.93)	7.3 (4.7, 11.4)	0.23 (0.14, 0.37)
4AT (ICU subgroup)	5 (1505)	0.76 (0.54, 0.89)	0.90 (0.78, 0.96)	7.38 (3.63, 15.01)	0.27 (0.13, 0.55)
4AT (non-ICU subgroup)	11 (2899)	0.82 (0.67, 0.91)	0.89 (0.81, 0.94)	7.36 (4.18, 12.96)	0.20 (0.11, 0.39)
AMT-4	3 (715)	0.93 (0.85, 0.97)	0.54 (0.48, 0.59)	2.02 (1.63, 2.36)	0.13 (0.06, 0.37)
MOTYB	5 (1537)	0.87 (0.83, 0.90)	0.61 (0.44, 0.76)	2.2 (1.5, 3.4)	0.22 (0.15, 0.30)
O3DY	1 (313)	0.84 (0.75, 0.91)	0.58 (0.52, 0.64)	2.01 (1.71, 2.37)	0.27 (0.17, 0.44)
UB-2	1 (293)	0.88 (0.72, 0.96)	0.61 (0.44, 0.76)	2.26 (1.28, 4.00)	0.20 (0.05, 0.64)

4AT, 4 attention test; AMT-4, 4-point abbreviated mental test; MOTYB, months of the year recited backwards; O3DY, Ottowa day, date, WORLD BW and Year; UB-2, ultra-brief 2-item screen; PLR, positive likelihood ratio; NLR, negative likelihood ratio.

**Figure 2:**
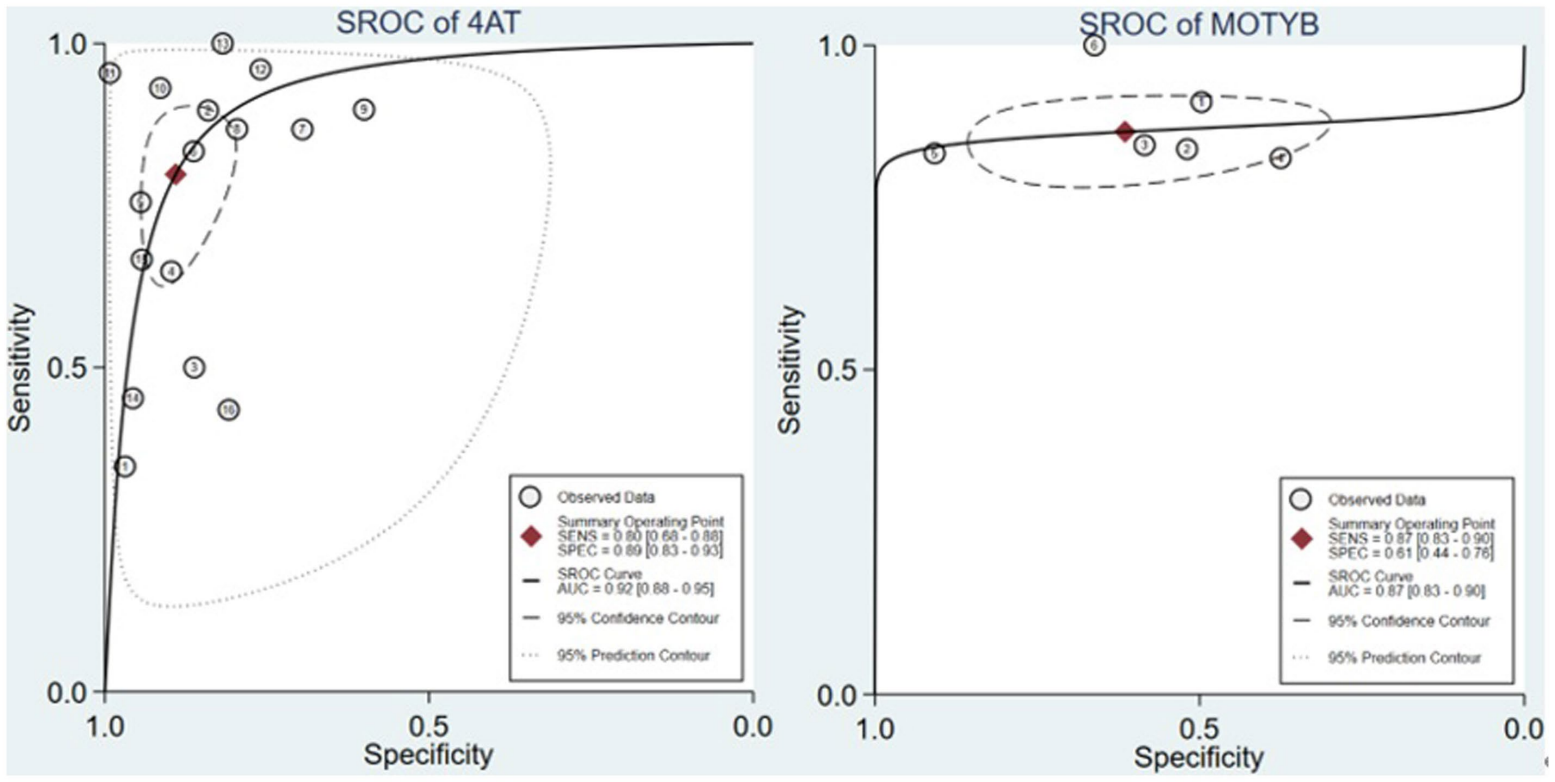
The SROC curves of 4AT and MOTYB.

The summary receiver operating characteristic (SROC) curves can eliminate the threshold effects of the instrument to predict overall accuracy. By the SROC curves of Figure 2, the 4AT had a higher AUC (*n* = 16 studies, AUC = 0.92) than MOTYB (*n* = 5 studies, AUC = 0.87). AMT-4, O3DY and UB-2 did not conduct SROC due to the lack of relevant research.

### Subgroup analysis

We performed a subgroup analysis of different sites (ICU or non-ICU) where 4AT was used. In the ICU, 4AT had a sensitivity of 76% (95% CI: 54%–89%) and a specificity of 90% (95% CI: 78%–96%); in the non-ICU, 4AT had a higher sensitivity of 82% (95% CI: 67%–91%) and a lower specificity of 89% (95% CI: 81%–94%). The PLR and NLR of the ICU were 7.4 (95% CI: 3.6–15.0) and 0.3 (95% CI: 0.1–0.6); those of the ICU were 7.4 (95% CI: 4.2–13.0) and 0.2 (95% CI: 0.1–0.4), respectively.

### Sensitivity analysis and publication bias

After the exclusion of non-DSM standard studies, the pooled sensitivity, specificity, PLR, and NLR for the 4AT were 80% (95% CI: 61%–92%), 88% (95% CI: 82%–92%), 6.5 (95% CI: 4.5–9.2), and 0.22 (95% CI: 0.10–0.48), respectively. MOTYB, AMT-4, O3DY and UB-2 did not conduct sensitivity analysis due to the lack of enough studies.

**Figure 3:**
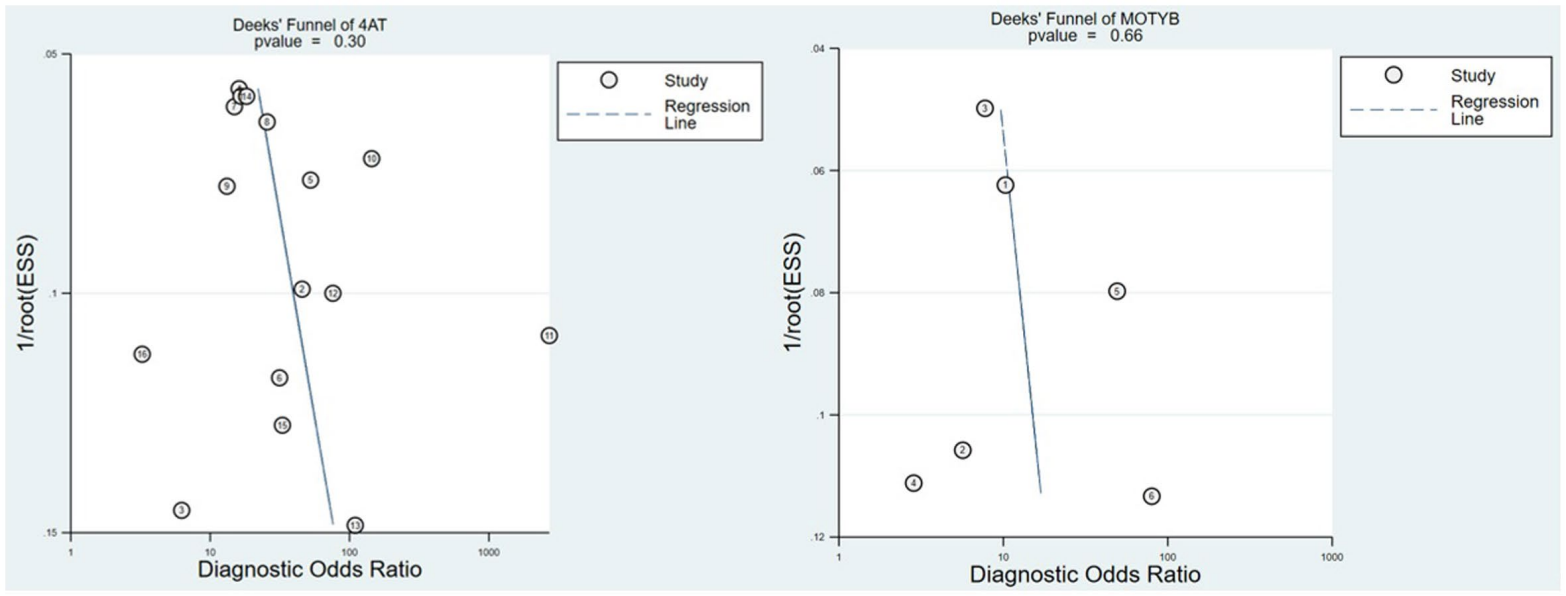
Deeks’ funnel plot of 4AT and MOTYB.

Deeks’ funnel plots revealed no evidence of publication bias, as shown in Figure 3 (4AT *p* = 0.3, MOTYB *p* = 0.66). We did not assess the publication bias of the AMT-4, O3DY and UB-2 because not enough studies were included.

## Discussion

Accurate recognition of delirium is clinically important to effectively provide clinical care and reduce late complications. To promote the detection rate of delirium, it is important to select appropriate methods and use them at least twice a day. Five instruments were included in our systematic review and showed that they may be used for multiple rapid screenings of delirium in clinical practice. The study quality of this meta-analysis was moderate to good overall, according to the QUADAS-2 assessment. Of the five screening tools, two instruments had sensitivity ≥80% and specificities ≥80%: 4AT and UB-2. These two instruments have unique strengths and limitations, and several potential scenarios for their use are provided here. Based on our recommended principles, we recommend 4AT as a clinical daily multiple rapid screening instrument.

The 4AT test includes two simple cognitive screening items. It is short (only 4 items and generally <2 min; Tieges et al., 2021), does not need special training, is easy to manage (including people with visual or hearing impairment), does not need physical response, and allows the evaluation of patients who “cannot be tested” (those who cannot be tested or interviewed due to severe sleepiness or excitement). 4AT has experienced several pilot rounds and has been used in many hospitals in the United Kingdom and internationally. The 4AT had a sensitivity of 80% and specificity of 89%, with a PLR of 7.3 and an NLR of 0.22. Although the 4AT has high sensitivity and specificity, it has the longest use time among the five scales included. There is a dynamic balance between performance and simplicity. Fortunately, we limited the ultrabrief scale when we included the article and then chose the best performance from it.

At present, there are few relevant studies on UB-2, which has only been verified in the United States. UB-2 is extracted from 3D-CAM (Fick et al., 2015), but the author does not recommend using UB-2 alone to diagnose delirium but uses the UB-CAM framework. Even UB-2 had a sensitivity of 88% and specificity of 64%, with a PLR of 2.4 and an NLR of 0.34. Another important item excluded by the author is “Does the patient report feeling confused?” That is, if these three items are positive, delirium can be directly diagnosed. More evidence of this screening tool is needed in the future.

Among the remaining five scales, MOTYB is the most studied. However, MOTYB, as a scale with only one test item, is extremely simplified in operation, but it has a low specificity of 61%. The five scales involved do not involve delusion, while a scale involving delusion, Nu-DESC, does not meet the criteria of the ultrasimple scale. The remaining three scales involved in this study have a common problem: there are too few original studies directly related to delirium, of which UB-2 lacks relevant studies due to its late launch.

Notably, AMT-4 itself is a part of the 4AT. Although the number of entries in the strict sense of the word is more than 4, in the practical application of the 4AT, the four questions about the AMT-4 can be asked in one question in one book,^1^ and it is not necessary to count the scores of each question but only the number of wrong answers, so it can be regarded as one item. This is different from using the RASS to evaluate the level of consciousness. RASS cannot be simplified into one problem (Ely et al., 2003).

This study has several advantages. First, we evaluated all screening tools’ COSMIN quality and evaluated the QUADAS-2 risk bias of the included studies. Second, we also followed the principle of a double review process and developed an evidence-based process for quality assessment. The methodological quality of the included studies was moderate to good overall. There have been many systematic evaluations of delirium screening instruments before (Wong et al., 2010), and they are constantly updated; however, this paper focuses on simplifying the instrument and achieving the screening effect as efficiently as possible.

There are several limitations to this study. First, the description of the use duration in each study is different, which is different from the actual use duration in other institutions. For this reason, after the description of the original literature and the actual simulation of the expert team, we have comprehensively set the duration and set it as the interval value after discussion. Second, many scales were designed for different user groups at the beginning of the design when the scale was included, so some scales had design defects, which led to poor final results and were finally eliminated. For example, the Delirium Triage Screen (DTS)/Brief CAM (b-CAM) itself was a simple enough screening strategy (Rieck et al., 2020), but the combination of the two parts exceeded the limit of items and was eliminated. This part of the scale should be classified and discussed in detail. Then, the evaluation of consciousness level in many scales is unclear (such as BCS). After we replace RASS, the number of items and operation time will be exceeded, and we have to abandon it. If there is a simpler way to assess the level of awareness, this part of the scale should also be included in the discussion. Finally, the scale recommended in this study is the 4AT. Although there is no language restriction, the scale included in this study is all in English, which obviously limits the strength of evidence for the use of the scale in other language regions.

This article provides an overview of the delirium scale that can be used for daily multiple screening in clinical work. Different assessors will choose different scales for screening in different clinical environments, but these scales may not be suitable for multiple use every day. This paper recommends a comprehensive and ideal scale “4AT,” which has a very high coverage of standard diagnostic criteria, which means that under ideal conditions, it can be used as the final diagnostic scale without requiring a professional doctor to diagnose. Moreover, because of the ultrasimple characteristics of 4AT, it can be used in clinical practice many times a day, which can reduce the delirium ignored due to the fluctuation of delirium, improve the detection rate, and ensure a good prognosis through early prevention.

In view of the high specificity of 4AT in the subgroup of nondementia patients and the high sensitivity of the subgroup of dementia patients, an important area of future research may be to improve the scale to improve its ability to identify delirium in dementia patients. It is hoped that the work of this paper will help improve the detection rate of delirium in clinical work and lay a foundation for promoting research in the field of delirium.

This study comprehensively summarized delirium screening tools based on the COSMIN guidelines. Five screening instruments were available, and the methodological quality assessment of the included studies by the QUADAS-2 tool was moderate to good. UB-2 and MOTYB had excellent sensitivity for delirium screening at an early stage. In terms of sensitivity and intentionality, the 4AT is the best recommended scale according to the results of this study.

## Author contributions

YaL and JY: study concept and design. YaL, ZL, and YiL: acquisition of data. YiL and YaL: analysis and interpretation of data. YaL and ZL: drafting of the manuscript. JY and NG: critical revision of the manuscript for important intellectual content. All authors contributed to the article and approved the submitted version.

## Funding

This work was supported by the National Key Research and Development Program of China (no. 2020YFC2005300), Sichuan Science and Technology Program (2022ZDZX0021; 2021YFS0139), 1.3.5 project for disciplines of excellence, West China Hospital, Sichuan University (ZYJC21005).

## Conflict of interest

The authors declare that the research was conducted in the absence of any commercial or financial relationships that could be construed as a potential conflict of interest.

## Publisher’s note

All claims expressed in this article are solely those of the authors and do not necessarily represent those of their affiliated organizations, or those of the publisher, the editors and the reviewers. Any product that may be evaluated in this article, or claim that may be made by its manufacturer, is not guaranteed or endorsed by the publisher.

## Footnotes

^1^ www.the4AT.com
