# Supplementary material for: Detecting delirium: a systematic review of ultrabrief identification instruments for hospital patients
Source: Front Psychol. 2023 May 12;14:1166392. doi: 10.3389/fpsyg.2023.1166392 (PMC10214704; doi:10.3389/fpsyg.2023.1166392)
Supplement: Supplementary file 1 [file Data_Sheet_1.docx]

**Supplement 1*:*** Database search strategy.

1. Database: Pubmed <1974 to 2022 December 31>

Search Strategy:

--------------------------------------------------------------------------------

| #1 | "Delirium"[MeSH] OR "Delirium"[tiab] OR "Acute confusion"[tiab] OR "Acute organic brain syndrome"[tiab] OR "Acute confusional state"[tiab] OR "Acute brain  syndrome"[tiab] OR "Acute brain failure"[tiab] OR “Acute brain dysfunction”[tiab] OR  "Acute organic psychosyndrome"[tiab] OR “Acute organic psycho-syndrome”[tiab]  OR "Acute psycho-organic syndrome"[tiab] OR “Acute psychoorganic syndrome”[tiab] OR "Metabolic encephalopathy"[tiab] OR “Clouded state”[tiab] OR “Clouding of consciousness”[tiab] |
| --- | --- |
| #2 | "Psychiatric Status Rating Scales"[MeSH] OR "Neuropsychological Tests"[MeSH]  OR "Psychometrics"[MeSH] OR "Mass Screening"[MeSH] OR "Geriatric  Assessment"[MeSH] OR "Psychological Tests"[MeSH] OR "Surveys and  Questionnaires"[MeSH] OR "Interview, Psychological"[MeSH] OR "Mental Status  Schedule"[MeSH] OR "Qualitative Research"[MeSH] OR "Checklist"[MeSH] OR  "Scale"[tiab] OR "Scales"[tiab] OR "Instrument"[tiab] OR "Instruments"[tiab] OR  "Measure"[tiab] OR "Measures"[tiab] OR "Questionnaire"[tiab] OR  "Questionnaires"[tiab] OR "Interview"[tiab] OR "Interviews"[tiab] OR  "Evaluation"[tiab] OR "Evaluations"[tiab] OR "Examination"[tiab] OR  "Examinations"[tiab] OR "Exam"[tiab] OR "Exams"[tiab] OR "Test"[tiab] OR  "Tests"[tiab] OR "Screening"[tiab] OR "Screenings"[tiab] OR "Assessment"[tiab] OR  "Assessments"[tiab] OR "Index"[tiab] OR "Indices"[tiab] OR "Indexes"[tiab] OR  "Qualitative Research"[tiab] OR "Qualitative Study"[tiab] OR "Qualitative  Studies"[tiab] OR "Checklist"[tiab] OR "Checklists"[tiab] |
| #3 | "Adult"[MeSH] OR "Young Adult"[MeSH] OR "Aged"[MeSH] OR "Aged, 80 and over"[MeSH] OR "Frail Elderly"[MeSH] OR "Adult"[tiab] OR "Adults"[tiab] OR "Young Adult"[tiab] OR "Young Adults"[tiab] OR "Middle age"[tiab] OR "Middle aged"[tiab]  OR "Elderly"[tiab] OR "Elder"[tiab] OR "Oldest old"[tiab] OR "Nonagenarian"[tiab] OR  "Nonagenarians"[tiab] OR "Octogenarian"[tiab] OR "Octogenarians"[tiab] OR  "Centenarian"[tiab] OR "Centenarians"[tiab] OR "Frail"[tiab] |
| #4 | "Alcohol withdrawal delirium"[MeSH] OR "Alcohol withdrawal syndrome"[tiab] OR  "Delirium tremens"[tiab] OR "Alcohol withdrawal delirium"[tiab] |
| #5 | "review"[Publication Type] OR "review literature as topic"[MeSH Terms] OR "systematic review"[All Fields] OR "meta-analysis"[Publication Type] OR "metaanalysis as topic"[MeSH Terms] OR "meta-analysis"[All Fields] |
| #6 | #1 AND #3 |
| #7 | #6 NOT #4 |
| #8 | #7 AND #5 |

**Supplement 2:**

COSMIN-guided Psychometric Review

Definitions and scoring approach:

The six reliability and validity criteria considered were:

1. Effect indicators: Effect indicators are influenced by or related to delirium, such as signs and symptoms of delirium. Effect indicators are appropriate for use in a measurement instrument. Cause or formative indicators are factors that might cause delirium (e.g., signs of infection), and would not be appropriate to include. Studies were given a score of 1 if all items were effect indicators or a score of 0 if the items included potential causative factors.

1. Content validity: refers to ensuring that all items capture relevant aspects of delirium. For instance, this can be assessed by face validity reviews involving experts, literature reviews, etc. If the study mentioned assessing content validity, then it was scored 1 otherwise failure to mention was scored 0.

1. Internal consistency: defines how each item relates to the others in the instrument. It is important to make sure the instrument assesses a single underlying construct, delirium identification. If the authors report internal consistency reliability with a value such as Cronbach’s coefficient alpha or McDonald’s omega coefficient, then a point was awarded.

However, if a sample size of less than 50 was used in calculating internal consistency they lose ½ point. If the authors failed to mention assessment of internal consistency, they were awarded no points.

1. Inter-rater reliability: refers to assessments of the agreement between two or more raters when making ratings on a single patient or research participant. We recorded any mention and statistics given including Pearson correlation coefficient, intra-class correlation coefficient, or Kappa statistics. If the authors mentioned inter-rater reliability, they were given a point and deduced a half point for using a sample size less than 50. They were given no points if they failed to mention any assessment of inter-rater reliability.

1. Construct validity: describes how well and instrument measuring a construct correlates with other instruments measuring the same construct, in this case delirium identification. If this comparison was performed, we recorded any correlation coefficients and awarded a point. We deducted a half point for using a sample size less than 50. They were given no points if they failed to mention any comparison instruments.

1. External (or criterion-related) validity: refers to comparison of the proposed instrument against a reference standard used for delirium case identification. We recorded the reference standard and awarded a point if assessed. We deducted a half point for using a sample size less than 50. They were given no points if they failed to mention any reference standard.
